# Using Exclusion-Based Sample Preparation (ESP) to Reduce Viral Load Assay Cost

**DOI:** 10.1371/journal.pone.0143631

**Published:** 2015-12-02

**Authors:** Scott M. Berry, Hannah M. Pezzi, Eram D. Williams, Jennifer M. Loeb, David J. Guckenberger, Alex J. Lavanway, Alice A. Puchalski, Cissy M. Kityo, Peter N. Mugyenyi, Franklin M. Graziano, David J. Beebe

**Affiliations:** 1 Department of Biomedical Engineering, University of Wisconsin - Madison, Madison, Wisconsin, United States of America; 2 Joint Clinical Research Centre; Kampala, Uganda; Institut National de la Santé et de la Recherche Médicale, FRANCE

## Abstract

Viral load (VL) measurements are critical to the proper management of HIV in developing countries. However, access to VL assays is limited by the high cost and complexity of existing assays. While there is a need for low cost VL assays, performance must not be compromised. Thus, new assays must be validated on metrics of limit of detection (LOD), accuracy, and dynamic range. Patient plasma samples from the Joint Clinical Research Centre in Uganda were de-identified and measured using both an existing VL assay (Abbott RealTime HIV-1) and our assay, which combines low cost reagents with a simplified method of RNA isolation termed Exclusion-Based Sample Preparation (ESP).71 patient samples with VLs ranging from <40 to >3,000,000 copies/mL were used to compare the two methods. We demonstrated equivalent LOD (~50 copies/mL) and high accuracy (average difference between methods of 0.08 log, R^2^ = 0.97). Using expenditures from this trial, we estimate that the cost of the reagents and consumables for this assay to be approximately $5 USD. As cost is a significant barrier to implementation of VL testing, we anticipate that our assay will enhance access to this critical monitoring test in developing countries.

## Introduction

The World Health Organization (WHO) guidelines for utilizing antiretroviral therapy (ART) in HIV infected individuals recommend initiating ART at a threshold CD4 count of 500 cells/mm^3^ or at any CD4 count when various confounding clinical factors are present [1]. It further strongly recommends HIV viral load (VL) measurement as the preferred method to monitor treatment once initiated [1,2]. In developed countries, VL monitoring to define ART success or failure is the standard of care. In most developing countries where income is low and the burden of HIV high, reliance upon CD4 measurement and clinical status monitoring is still the norm. Many studies in adults and children initiating ART and living in low income countries now report that the strategy of evaluating CD4 count and clinical status without VL monitoring leads to unnoticed virologic failure [3].

By the end of 2012, 9.7 million individuals infected with HIV were receiving ART in developing countries [4]. However, the WHO reports only 1.2 M VL tests were performed annually in the 66 reporting countries. It has been further estimated that approximately 23% of the people receiving ART in Africa receive routine VL testing [1,5]. While VL testing critically measures the number of copies of HIV in the blood, the effectiveness of ART therapy, and treatment failure prior to the onset of clinical symptoms, a number of recognized challenges to implementing routine VL monitoring in developing countries exist. Important among these are complexity of the instrumentation for the test, need to bring the test closer to the site of patient care, and cost [4]. This report describes studies performed in Uganda utilizing Exclusion-Based Sample Preparation (ESP). ESP targets the challenges described by utilizing a method for nucleic acid isolation that is simple, rapid to perform, and electricity-free[6,7]. ESP can be performed manually or can be automated on a robust, low cost open-architecture system for higher throughput [8–10]. Additionally, these simplifications result in significant cost savings over current nucleic acid analysis techniques and may enhance access to viral load testing, particularly in low-income areas including the epicenter of the HIV epidemic.

## Materials and Methods

### Device Fabrication

**Fig 1:**
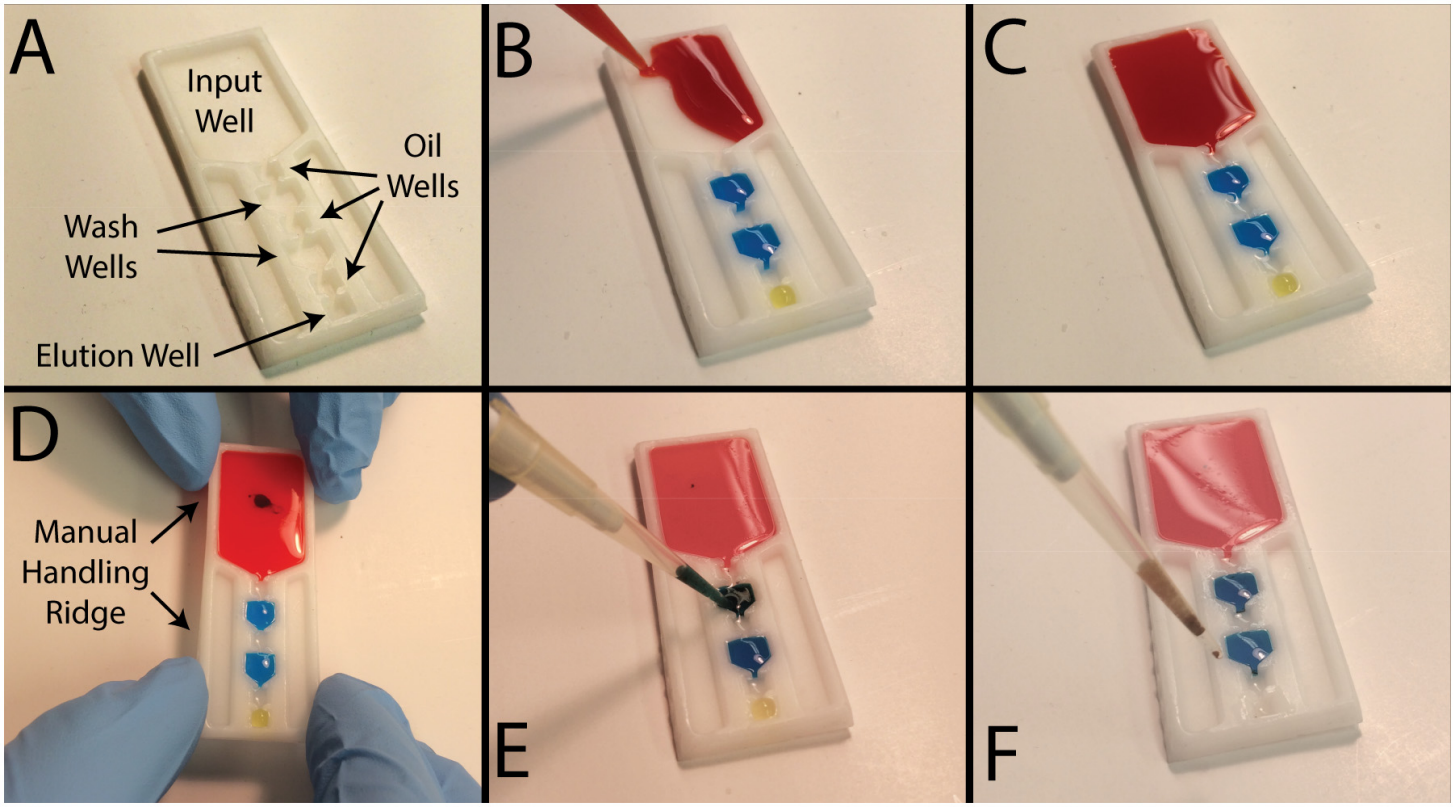
Loading and operation of ESP devices. A) An unfilled device with each well labeled. B) Aqueous reagents are first added to the ESP device. C) Oil is added last, between each aqueous reagent. D) Manually operated devices are held by ridges located around the periphery of the ESP device to prevent contact between the reagents and the operator’s hand. E) Optionally, PMPs can be mixed within each wash well to enhance purity. F) After operation, the eluent is removed from the ESP device via pipette.

ESP devices (Fig 1A) were embossed from paraffin wax (Sasol Wax GmbH) as previously described [7,8]. Briefly, a petri dish of wax was heated above its melting point (to approximately 140C) and allowed to cool until solidification began to occur. An aluminum ESP mold machined using a CNC milling machine (Tormach) was manually pressed into the soft, semi-solid wax and cooled for five minutes to allow complete solidification. The mold and wax were separated, resulting in a casting of wax ESP devices.

### Device Operation

For each sample, 200 μL of patient plasma was mixed with 800 μL of lysis buffer (mLysis, MD130A), 40 μL of paramagnetic particles (PMPs; mMicroparticles, MD134A) that are functionalized to capture viral RNA, 5 μL of food dye (McCormick, used as a rapid indicator of eluent purity; carryover of visible amounts of dye indicates a failed device), and 17.8 μL of internal control (Abbott RealTime HIV-1 Internal Control) and heated to 50C for 20 minutes to promote viral lysis. This lysate was loaded into the input well of an ESP device and 60 μL of each wash buffer (mWash 1, MD131A; mWash 2, MD132A, both from Abbott) and 20 μL of elution buffer (mElution Buffer, MD133A, Abbott) were added to the appropriate wells (Fig 1B; see Fig 1A for reagent well locations). Once all aqueous buffers were added, oil (FC40, Sigma-Aldrich) was added between each aqueous well (40 μL of oil between the two wash buffers, 20 μL of oil for all other locations; Fig 1C) to serve as a hydrophobic barrier between aqueous solutions.

To operate the ESP devices, a magnet was slid beneath the devices to pull the PMPs (with viral RNA attached) through the wash buffers and oil-filled wells (Fig 1D). ESP devices were operated completely manually (held by hand) or using a custom-made apparatus that aligns the magnet and devices (S1 Fig). This jig allows a user to load three ESP devices into a holder, which is then loaded onto a base with a magnetic slider that can be actuated to operate all three devices in parallel. In both the complete manual and jig configurations, magnets were initially located under the input well of the device and slowly (approximately 1–2 mm/s) drawn toward the elution buffer. Magnetic actuation was paused as the PMPs passed through each wash buffer and a pipette was used to briefly (~5 seconds) mix the PMP aggregate (Fig 1E, S1 Video). These two mixing steps were added to enhance the purity of the viral RNA. PMPs were collected from the elution well via pipette (Fig 1F), placed in a tube and heated to 75C for 20 minutes to promote viral RNA elution. An additional 63uL of Wash Buffer 2 was then added to the eluent. PMPs were magnetically drawn to the side of the elution tube and the supernatant was collected, which was then quantified as described in the next section. It should be noted that analysis was also performed without removing the PMPs with minimal influence on quantitation, but PMP removal was included here to minimize non-ESP differences in this comparison trial. Overall, the ESP isolation process requires less than 1 minute of hands-on time per sample, a value that is further reduced by loading and operating multiple ESP devices in parallel.

### Comparison Trials

To evaluate the effectiveness of ESP-based viral RNA isolation, a trial was constructed to compare ESP extraction performance with a “gold standard” viral RNA isolation instrument (Abbott m2000SP). In this trial, RNA was extracted from 71 excess plasma samples collected from patients at the Joint Clinical Research Centre (JCRC) in Kampala, Uganda. These samples were collected via a protocol approved by the institutional review boards (IRBs) of the University of Wisconsin—Madison, the JCRC, and the Ugandan Ministry of Health. These IRBs waived the need for patient consent as samples were analyzed anonymously using excess plasma from other clinical testing. Samples were split, and RNA was isolated using both ESP (as previously described) and the standard instrument (following the manufacturer’s protocol for 0.6 mL samples). Following isolation, both sets of RNA were simultaneously amplified on a single plate using the Abbott HIV-1 RealTime Assay reagents as previously described [11]. Furthermore, the Abbott m2000RT thermocycler (using the Abbott HIV-1 RealTime Assay cycling conditions) was used for both the Abbott and ESP sets of RNA, as to not convolute the assay comparison with differences in cycler performance. Viral load measurements were adjusted to account for discrepancies in the amount of sample processed with each method (0.2 mL with ESP vs. 0.6 mL with the standard method).

In order to minimize the cost of the complete assay, we replaced the Abbott RT-qPCR reagents with a “generic” option previously reported by Rouet *et al*. [12]. While this lower cost RT-qPCR assay was extensively validated with patient samples during the original report, we wanted to confirm its compatibility with ESP-purified RNA. Thus, 32 additional comparison trials were run on ESP-purified RNA to compare the performance of the low cost amplification reagents with the “gold standard” Abbott reagents. Specifically, the PCR primers and probe (FAM) specified by Rouet *et al*. and Veronique *et al*. [12,13] (synthesized by Life Technologies and specific to the LTR region of HIV) were mixed with RNA and a commercially available RT-PCR master mix (Taqman Fast Virus One-Step MasterMix, Life Technologies) at concentrations of 1.5 μL primer/probe mix and 12.5 μL of 4X MasterMix in a total reaction volume of 50 μL (the primer and probe sequences were as follows: forward primer: 5′-GCCTCAATAAAGCTTGCC-3′; reverse primer: 5′-GGCGCCACTGCTAGAGATTTT-3′; probe: 5′-AAGTAGTGTGTGCCC-3′). Additionally, RNA was extracted from breast cancer epithelial cells transfected to stably express enhanced green fluorescent protein (eGFP; generous gift of Dr. Elaine Alarid, who transfected MCF-7 cells (ATCC) to express eGFP). This RNA was used as a low cost internal control (eGFP is not expressed in humans or HIV) since additional eGFP RNA can be easily produced at very high concentrations from cultured cell lines. The eGFP RNA was co-amplified using a commercially available primer / probe (VIC) mixture (Life Technologies Product #Mr04329676_mr). Reference RNA was spiked into samples at a concentration of 10,000 cycles per mL of sample. Empirically, we determined that this level of internal control resulted in detectable amplification in an average of 21.5 cycles. Based on this information, we chose to reject all ESP-isolated RNA that produced threshold cycle values in excess of 23, which is consistent with levels used by the gold standard (Abbott) system. In addition, reference samples (200 cp/mL virology quality assurance (VQA) standards diluted in negative serum to 50 cp/mL) were run 10 times to evaluate the limit of detection (LOD) of the ESP-based protocol.

### Analysis of Data and Costs

To compare experimental conditions, quantitation cycles were obtained for each run and converted to viral load using the equation:

$$Viral\;Load=\;e^{\frac{C_{Q}-32.1}{-1.42}}$$

where C_Q_ is the quantitation cycle of the RT-qPCR reaction. This equation was determined by fitting a curve to a plot of viral load vs. C_Q_ for known samples. Scatter plots were generated for each comparison and coefficients of determination (R^2^) were calculated to determine if a linear correlation exists between the data sets. In addition, Bland-Altman plots were constructed to analyze agreement between data sets. To estimate the projected total cost of the viral load assay, we examined the current cost structure for viral load assays at the JCRC. Using these values as a starting point, we identified costs associated with the reagents and equipment that may be affected when implementing ESP and/or low cost RT-qPCR reagents. Using high volume retail prices to estimate the costs of the replacement reagents, we then calculated the new viral load assay cost projection.

## Results

### Comparison of ESP-Isolated RNA with Gold Standard RNA Isolation

**Fig 2:**
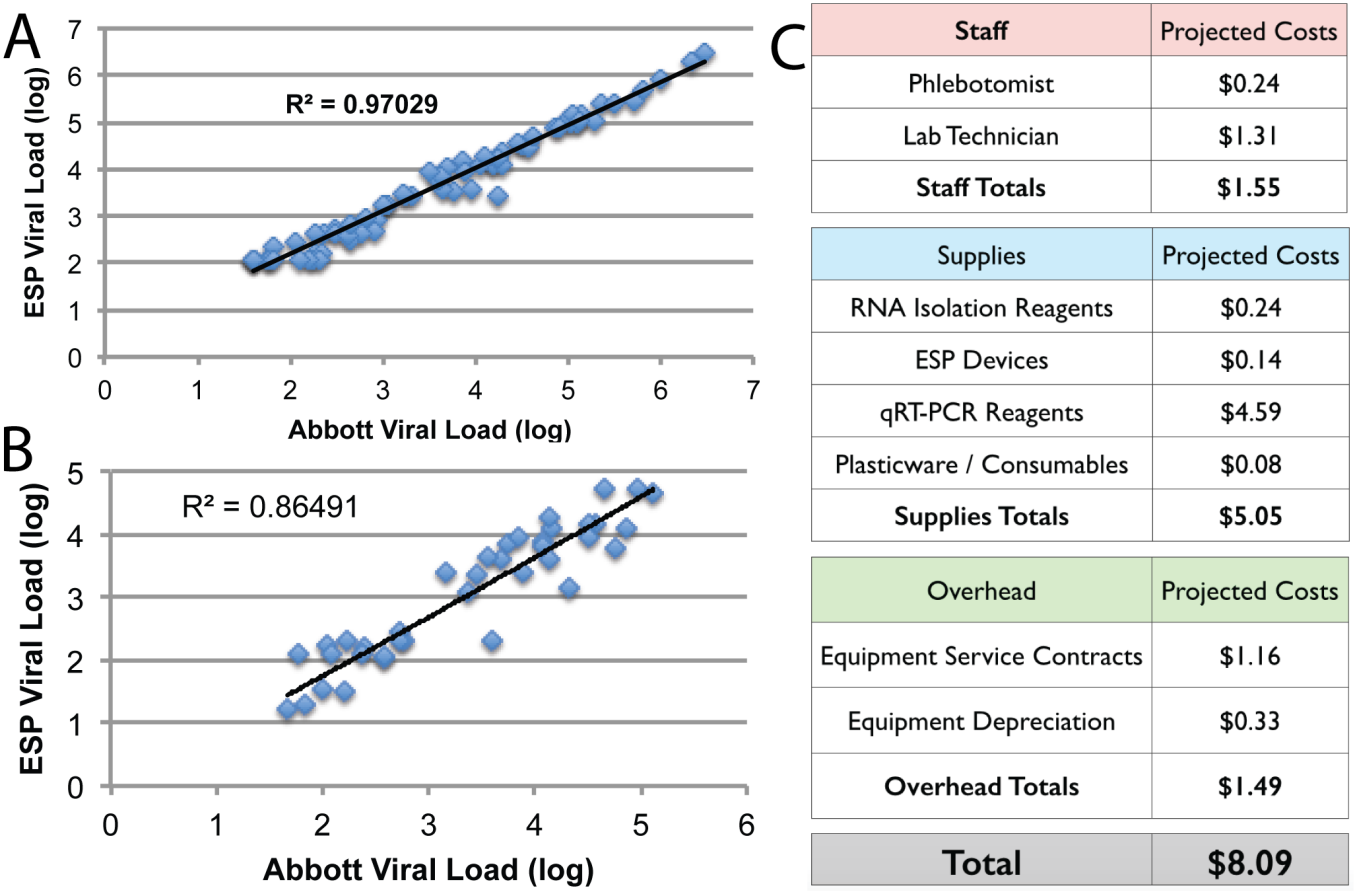
Results of the ESP comparison trial. A) Comparison of RNA extraction between ESP and gold standard protocols; B) Comparison of low cost RT-qPCR reagents with gold standard reagents on ESP-extracted RNA; C) Breakdown of total assay costs for ESP / low cost reagent protocol.

A total of 71 HIV+ plasma patient samples were obtained from the JCRC and RNA was extracted using both ESP and Abbott m2000sp sample preparation. JCRC lab technicians initially evaluated both completely manual operation and “jig operation” (shown in S1 Fig), and chose to focus on the completely manual process since no handling or operation problems were observed with this configuration and this process involved fewer steps (and less time) than the jig configuration. Over the course of this study, five ESP samples were excluded from analysis due to out-of-spec internal control signal (as comparison, 16 Abbott samples failed due to erroneous liquid handling) and samples that failed with either method were excluded from the remainder of the analysis. Purified RNA was amplified and quantified using the Abbott HIV-1 RealTime RT-PCR system. Patient sample viral loads ranged from <40 cp/mL to 3,000,000 cp/mL. A comparison of the viral loads (Fig 2A) confirms excellent correlation between the two RNA extraction methods (R^2^ = 0.97, slope = 0.92, y-intercept of 2.3 cp/mL). In addition, two samples (both <40 cp/mL) were detected from the Abbott purified RNA that were not detected from the ESP purified RNA. Samples measuring <40 cp/mL are generally considered to be “undetectable” (even though they were qualitatively identified via the Abbott assay) and are indicative of successful ART treatments. Furthermore, the lack of detection with ESP may be due to the reduced sample volume for ESP (200 μL) compared with the Abbott system (600 μL). The mean difference between the ESP and Abbott datasets was 0.08 log. A Bland-Altman plot of this data (S2 Fig) illustrates that the ESP values will lie within 1.9 fold (0.28 log) of the Abbott values (within 95% confidence interval). To quantify the limit of detection, reference samples containing 50 cp/mL were quantified ten times with the ESP protocol and virus was detected at a rate of 70%, which compares favorably to a reported 68% detection rate with the Abbott system for identical sample volume (200 μL) and virus concentration [11].

### Comparison of Low Cost RT-PCR Reagents to Gold Standard Reagents

To further reduce total assay costs, we next compared the performance of the gold standard RT-PCR reagents with a low cost alternative that has been previously validated in clinical settings in the developing world. Although these reagents have been previously validated, we benchmarked their performance against the gold standard reagents with an additional 32 patient samples. Again, comparison of the viral loads (Fig 2B) confirms excellent correlation between the two reagent mixtures (R^2^ = 0.88, slope = 0.92, y-intercept of 1.0 cp/mL), even when comparing 25 μL total RT-PCR reaction volume with the low cost reagents with 50 μL of total volume with the gold standard reagents. In addition, we successfully detected our eGFP mRNA-based internal control in all samples with high stability. The low cost internal control was detected at an average of 21.5 PCR cycles with a standard deviation of 0.7 cycles, compared with 21.1 ± 0.3 cycles for the gold standard internal control. Together, these results demonstrate that total assay cost can be further reduced by combining ESP RNA isolation with lower cost RT-PCR reagents.

## Discussion

We have successfully demonstrated a viral load assay that performs equivalently to the gold standard on metrics of accuracy, limit of detection, and range. A goal of this study was to decrease the cost of VL without diminishing performance, thus improving access to this critical test at the epicenter of the HIV/AIDS epidemic. As a component of this study, costs were tracked for the ESP-based assay including reagent costs, device costs, labor costs, instrument costs, and overhead costs. Reagent and materials costs were set as the retail prices paid to suppliers (which could likely be reduced through high volume purchasing) and labor, instrument, and overhead costs were estimated by the JCRC based on their current rates for these expenditures. A summary of costs is given as Fig 2C, illustrating that an ESP-based VL has the potential to reduce assay costs to approximately $5 USD for reagents and consumables. Based on current costs at the JCRC clinics, we estimate that the total assay cost will be approximately $8 USD including $1.50 USD each for labor and equipment costs.

A recently published cost effectiveness study demonstrated that adding routine (4X per year) monitoring of VL to clinical and CD4+ monitoring has an incremental cost effectiveness ratio (ICER) of $5,181 per disability adjusted life year (DALY) averted in Uganda [14]. This study concluded that, at current VL costs, VL was *not* cost effective despite reductions in mortality and morbidity. Another study in Cameroon [15] drew the same conclusion for a “gold standard” VL assay, but suggested that a lower cost VL test may be considered cost effective. In this study, “cost effectiveness” was defined using the WHO‐established cost effective guideline of 3 times GDP per capita [16]. Applying this guideline to Uganda, an intervention in Uganda must cost less than $1,746 per DALY in order to be considered cost‐effective. Furthermore, Boyer *et al*. reported that quarterly VL testing averted 0.1 DALYs per year. Thus, using the costs shown in Fig 2C and previously reported ART costs [15], we project a cost of $621 per DALY averted when employing ESP and low cost RT-PCR reagents.

In summary, we have developed and tested an HIV viral load assay with a cost that is less than current “gold standard” VL assays. These savings were realized through simplification of the operational protocol and substitution of lower cost, but extensively validated, reagents. In addition, field-testing at the JCRC demonstrated the ability to train lab staff with our assay and achieve results that are comparable to those obtained with the gold standard assay. As a next step, we plan to implement this assay in a remote clinic with moderate infrastructure (e.g., electricity, lab technicians with general training, access to -20C storage) that currently lacks access to viral load testing. We plan to evaluate both the manual version reported here as well as a low cost automated version. This pilot implementation is a step towards the goal of providing access to viral load assays across all of Sub-Saharan Africa.

## Supporting Information

S1 Fig Photos illustrating construction and operation of “jig” used to operate ESP devices including A) holder for three ESP devices and B) base including magnetic slider. C) Operation involves loading the holder onto the base and sliding the magnets beneath the ESP devices.
(JPG)

S2 Fig Bland-Altman plot illustrating 95% confidence interval of ESP-based VL assay compared to gold standard (assuming 100% accuracy for gold standard assay). (TIF)

S1 Video Loading and operation of an ESP device. (MP4)
