# Supplementary figures and images for: Using Exclusion-Based Sample Preparation (ESP) to Reduce Viral Load Assay Cost
Source: PLoS One. 2015 Dec 2;10(12):e0143631. doi: 10.1371/journal.pone.0143631 (PMC4667969; doi:10.1371/journal.pone.0143631)

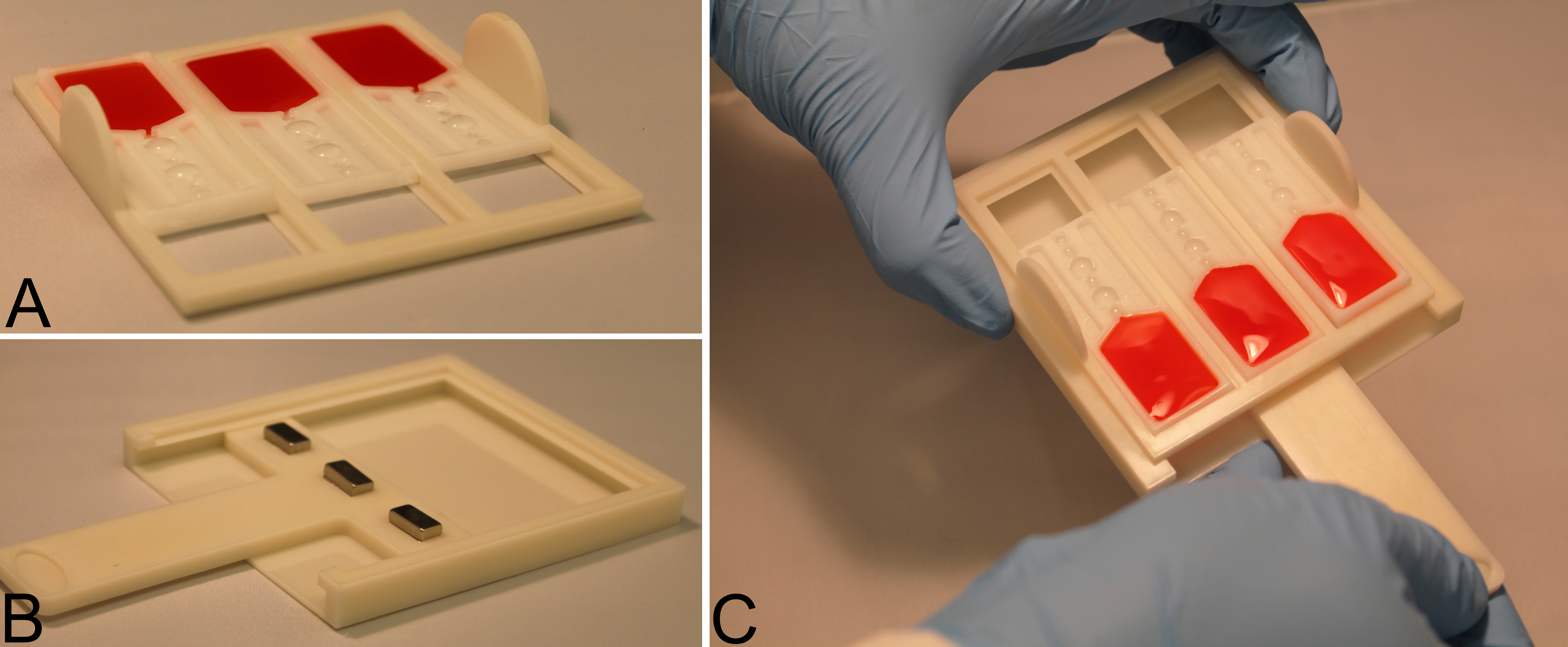

Supplement: S1 Fig — Photos illustrating construction and operation of “jig” used to operate ESP devices including A) holder for three ESP devices and B) base including magnetic slider. C) Operation involves loading the holder onto the base and sliding the magnets beneath the ESP devices. (JPG) [file pone.0143631.s001.jpg]

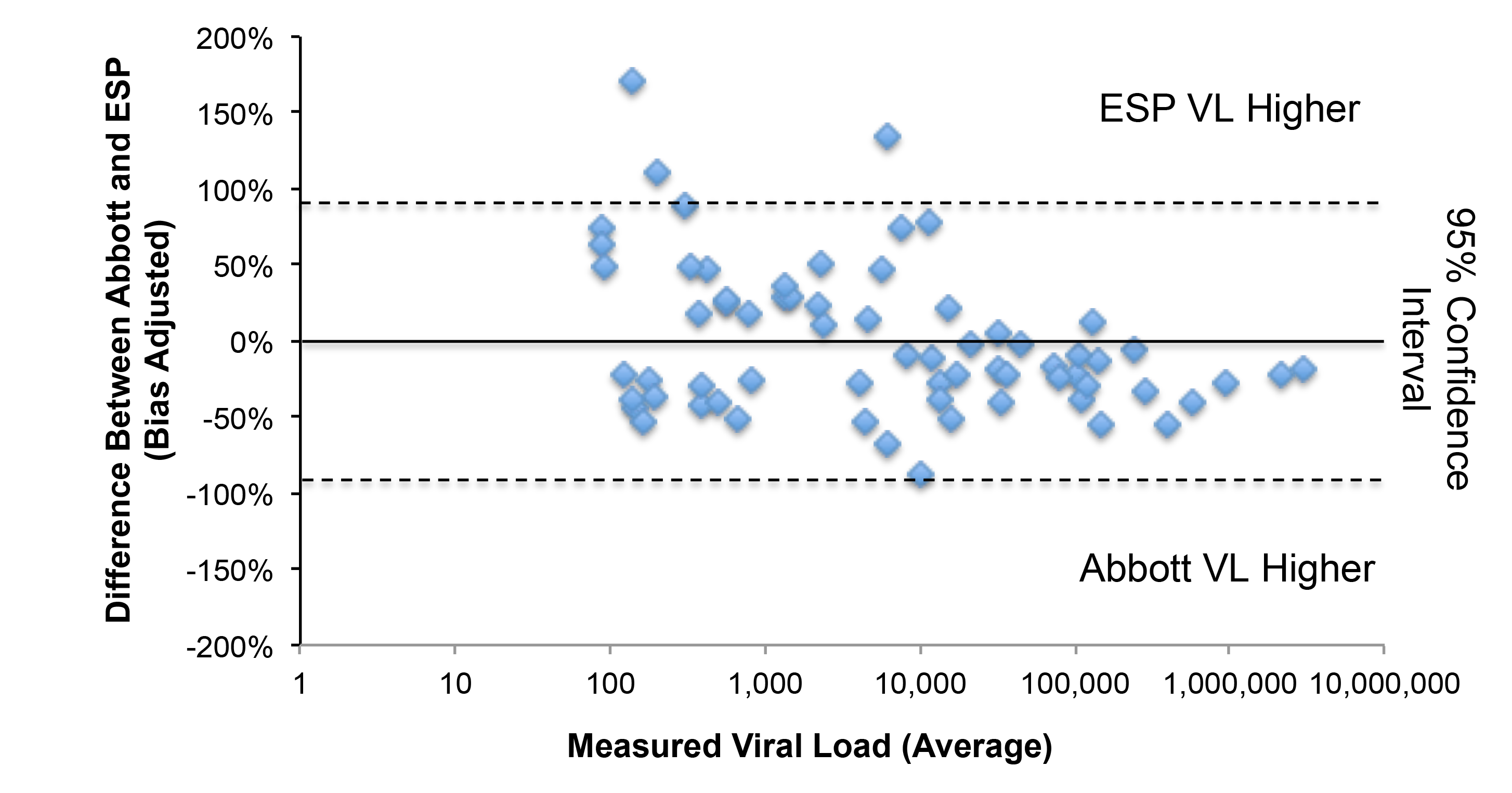

Supplement: S2 Fig — (TIF) [file pone.0143631.s002.tif]
